# Occult replication of a conditionally-live attenuated SIV profoundly upregulates T effector memory cell frequency

**DOI:** 10.1186/1742-4690-8-S2-O41

**Published:** 2011-10-03

**Authors:** Maria Manoussaka, Richard Stebbings, Neil Berry, Atze Das, Ben Berkhout, Neil Almond, Martin Cranage

**Affiliations:** 1 Centre for Infection and Immunity, St George's, University of London, London, SW17 ORE, UK; 2 Division of Retrovirology, National Institute for Biologica Standards and Control, Potters Bar, EN6 3QG, UK; 3 Laboratory of Experimental Virology, Academic Medical Center of the University of Amsterdam, Amsterdam, The Netherlands

## Background

The most potent protection against infection with virulent SIV, including protection against mucosal challenge, is conferred by "vaccination" with live attenuated virus. Although this approach is precluded for HIV because of safety concerns, understanding the mechanisms of superinfection resistance may inform rational vaccine design.

## Materials and methods

In order to uncouple antigen exposure from active viral replication we compared peripheral and intestinal T cell phenotypeand SIV peptide-specific responses following infection of macaques with wild type SIVmac239, attenuated SIVmac239Δ*nef* or with a doxycycline (dox)-conditional replication variant of SIVmac239 ∆*nef* designated SIVrtTA. Global (antigen-non-specific) T cell phenotype was assessed for central memory (Tcm) (CD28^+^CCR7^+^CD95^+^), transitional (CD28^+^CCR7^-^CD95^+^) and effector memory (Tem) (CD28^-^CCR7^-^CD95^+^) and SIV-specific T cell responses were measured by detection of TNF-α, IFN-γ, IL-2 and IL-17.

## Results

In animals in which on-going virus replication was permitted, albeit not detectable in the systemic circulation, (SIVrtTA + dox & SlVmac239Δ*nef*), the proportion of CD4 and CD8 Tcm and transitional T cells were reduced in the majority of animals while Tem proportions increased. In animals infected with SIVrtTA in which dox had been withdrawn for 8 weeks prior to analysis, these changes were not seen. Moreover animals infected with wild type virus had elevated CD4 and CD8 Tcm. Analysis of gut mucosal homing (α4^+^β7^+^and β7^+^)T cells showed similar polarised changes.

## Conclusions

Overall, we found that active occult replication of SIVrtTA and SIVmac239Δ*nef* had a profound impact on global T cell phenotype and antigen-specific polyfunctionality in the periphery and the gut. The use of these SIV mutants will contribute to the understanding of the mechanisms of superinfection resistance.

